# Introgressions of Vitis rotundifolia Michx. to obtain grapevine genotypes with complex resistance to biotic and abiotic stresses

**DOI:** 10.18699/VJ21.079

**Published:** 2021-11

**Authors:** V.A. Volynkin, V.V. Likhovskoi, I.A. Vasylyk, N.A. Rybachenko, E.A. Lushchay, S.M. Gorislavets, V.A. Volodin, V.I. Risovannaya, E.K. Potokina

**Affiliations:** All-Russian National Research Institute of Viticulture and Winemaking “Magarach” of the Russian Academy of Sciences, Yalta, Russia; All-Russian National Research Institute of Viticulture and Winemaking “Magarach” of the Russian Academy of Sciences, Yalta, Russia; All-Russian National Research Institute of Viticulture and Winemaking “Magarach” of the Russian Academy of Sciences, Yalta, Russia; All-Russian National Research Institute of Viticulture and Winemaking “Magarach” of the Russian Academy of Sciences, Yalta, Russia; All-Russian National Research Institute of Viticulture and Winemaking “Magarach” of the Russian Academy of Sciences, Yalta, Russia; All-Russian National Research Institute of Viticulture and Winemaking “Magarach” of the Russian Academy of Sciences, Yalta, Russia; All-Russian National Research Institute of Viticulture and Winemaking “Magarach” of the Russian Academy of Sciences, Yalta, Russia; All-Russian National Research Institute of Viticulture and Winemaking “Magarach” of the Russian Academy of Sciences, Yalta, Russia; All-Russian National Research Institute of Viticulture and Winemaking “Magarach” of the Russian Academy of Sciences, Yalta, Russia; Saint Petersburg State Forest Technical University, St. Petersburg, Russia

**Keywords:** grapes, Vitis vinifera L., Vitis rotundifolia Michx., backcrosses, biotic and abiotic stress, powdery mildew, frost, resistance, genes, introgression, виноград, Vitis vinifera L., Vitis rotundifolia Michx., беккроссы, биотический и абиотический стресс, мучнистая роса, мороз, устойчивость, гены, интрогрессия

## Abstract

Vitis rotundifolia Michx. is one of the species of the family Vitaceae, with resistance to both biotic and abiotic stresses. The present study reports new scientif ic knowledge about the inheritance of resistance to downy mildew, powdery mildew and frost by V. vinifera varieties from V. rotundifolia. Recombinant lines of three hybrid populations from the crossing of the maternal genotype ♀M. 31-77-10 with V. rotundifolia hybrids were used as the object of the study. As a result of laboratory screening, more than 40 % of recombinants of the ♀M. 31-77-10 × × [DRX-M5-734 + DRX-M5-753 + DRX-M5-790] population showed a high degree of frost resistance (–24 °C), while 6 % of transgressive recombinants were characterized by a very high degree of resistance (–27 °С). The maternal genotype ♀M. 31-77-10 does not carry alleles of resistance to powdery mildew at the Run1 locus and in the f ield suffers from powdery mildew much more than the paternal genotypes. The prevalence of powdery mildew on vegetative organs in the three recombinant populations over the years varies on average between 3.2–17.1, 0.3–17.7 and 0.6–5.2 %, respectively. As a result, almost all recombinant genotypes that received a resistant allele from the paternal genome are highly resistant to powdery mildew.

## Abstract (ru)

Vitis rotundifolia Michx. – один из видов в семействе Vitaceae, демонстрирующий устойчивость как к биотическим, так и к абиотическим стрессам. В процессе изучения получены новые научные знания о наследовании культурным виноградом от V. rotundifolia признаков устойчивости к патогенам, вызывающим милдью и оидиум, и к морозу. Объектом исследования служили рекомбинантные линии трех популяций от скрещивания материнской формы ♀M. 31-77-10 с гибридами потомства V. rotundifolia. Установлено, что признак морозостойкости, скорее всего, имеет полигенное наследование. По результатам лабораторного изучения, в популяции ♀M. 31-77-10 × [DRX-M5-734 + DRX-M5-753 + DRX-M5-790] более 40 % рекомбинантов характеризуются высокой степенью морозоустойчивости (–24 °C), в то время как 6 % трансгрессивных рекомбинантов – очень высокой степенью устойчивости (–27 °C). Материнский генотип ♀M. 31-77-10 не несет аллелей устойчивости к оидиуму в локусе Run1 и сильнее, чем отцовские генотипы, поражается оидиумом в полевых условиях. Распространение оидиума на вегетативных органах в трех рекомбинантных популяциях в среднем за годы исследований колеблется в пределах 3.2–17.1, 0.3–17.7 и 0.6–5.2 % соответственно. Почти все рекомбинантные генотипы, получившие аллель устойчивости в локусе Run1 от отцовского генома, обладают высокой устойчивостью к оидиуму

## Introduction

Remote hybridization plays an important role in modern grape breeding. It allows combining in hybrid progeny traits of various Vitis species, which have significantly diverged in evolution; for example, high productivity and high berries quality of the Vitis vinifera L. varieties with resistance to biotic and abiotic stress of American Vitis species. Hybridization makes it possible, on the one hand, to obtain experimentally new forms and varieties, on the other hand, to study the relationship between genomes, structure and function of chromosomes, the patterns of inheritance of morphological and economically valuable traits. N.I. Vavilov (Vavilov, 1986) emphasized that employing remote hybridization is especially promising for the breeding of vegetative propagated plants, including grapes

Significant success was achieved by grape breeders and growers in the development of interspecific hybrids and in the study of such important issues as the selection of parental pairs, dominance, coping with incapacity for hybridization, identifying the sources of inter-sterility and reduced fertility of hybrid plants. In contrast to the V. vinifera L. cultivars, many other Vitis species, native to North and Central America, especially V. rotundifolia, are distinguished by high resistance to pathogens, pests and frost. Therefore, breeders and grape growers have always found the creation of new cultivated varieties of grapes promising, combining productivity and quality of V. vinifera with resistance of American Vitis species, meaning to create a “perfect” grape variety. In the middle of the 20th century in Europe there even existed a “perfect variety” breeding program, which has been transformed in the ‘Magarach’ Institute into the breeding program “Analogue” (Volynkin et al., 2018). Currently, this breeding program has found its further development in the introgression of V. rotundifolia genes into V. vinifera genome (Volynkin et al., 2020а). It should be noted that the Institute of Viticulture and Winemaking ‘Magarach’ is one of the leading centers of grape breeding in the world (Volynkin et al., 2015), and its grapevine breeding program is based on the study of the world Vitis gene pool and international trends of viticulture (Volynkin et al., 2021a).

The significance of such a breeding program is explained by the fact that a considerable part of vineyards in the Russian Federation is located in the zone of risky viticulture and almost every year suffers from frost coupled with the intensive development of downy mildew (caused by Plasmopara viticola Berl. et De Toni) and powdery mildew (Erysiphe necator Schwein.). In these conditions, the period of growing season of grape plants is reduced. Besides, in winter, plants are exposed to temperatures lower than the biological adaptive capacity of this species allows.

The study of the inheritance of grape frost resistance in progeny made it possible to establish that the trait is determined, first of all, by biological specificity of a grape genotype. Some Vitis species die in mild frosts; others are able to survive in the most severe winters (Likhovskoi et al., 2019; Vasylyk et al., 2020). Frost resistance is also influenced by soil and climatic conditions as well as agrotechnical methods that provide plants with optimal conditions for nutrition, water supply and airing. Cultivated grapevine in natural field conditions usually do not achieve maximum frost resistance, since the conditions of their preparation for the winter period are often unfavorable (Pavloušek, Postbiegl, 2003; Xiaoyan et al., 2015; Polulyakh et al., 2017).

Diagnostics of the frost resistance of grape varieties plays an important role in breeding, because only if information about the degree of a trait assigned to a particular genotype is complete and accurate, it can be used as a source of a valuable trait in breeding (Kozma, 1998; Korbuly, 2000; Clark, Barchenger, 2015; Ivanisević et al., 2015; Gonçalves et al., 2016; Volynkin et al., 2020b, с). In modern research, scientists are searching for the ways of conducting express-diagnostics of the frost resistance degree based on correlations with morphological traits (Maltabar, Zhdamarova, 2012; Novikova, Naumova, 2018; Ilnitskaya et al., 2019; Volynkin et al., 2020d), or studying biochemical mechanisms of the resistance and adaptation of grape plants to environmental stress factors at the molecular level (Di Gaspero et al., 2007; Nenko et al., 2019; Ricciardi et al., 2021; Shen et al., 2021). The most complete and reliable information about the resistance of grape varieties to environmental stress factors can be obtained only as a result of combination of field and laboratory experiments (Korbuly et al., 2004; Read et al., 2004; Ulitin, Nudga, 2008; Zlenko et al., 2018).

The development of new grapevine varieties that ensure ecological purity of food based on genetically determined resistance to pathogens in combination with frost resistance is one of the priorities in modern grape breeding.

## Materials and methods

Plant material. The studies were carried out in 2017–2020 in field and laboratory conditions. The object of the study was the recombinant lines of three populations obtained in the ‘Magarach’ Institute from the following crosses: ♀M. 31-77-10 × × [DRX-M5-734 + DRX-M5-753 + DRX-M5-790] (66 hybrids), ♀M. 31-77-10 × 2000-305-143 (43 hybrids) and ♀M. 31-77-10 × 2000-305-163 (30 hybrids). Hereinafter, they are referred to as populations 2-11, 3-11 and 4-11, respectively. The maternal genotype ♀M. 31-77-10 was obtained at the ‘Magarach’ Institute by crossing the cv. Nimrang (V. vinifera) with Seibel 13666 (a complex interspecific hybrid). In turn, the two paternal genotypes are progeny of the NC16-5 (V. rotundifolia × V. vinifera) backcrosses with various varieties of V. vinifera. To ensure the greatest reliability of the crosses performed, the maternal genotype taken for crossing possessed a functionally female type of flower, excluding the possibility of self-pollination.

Climatic conditions. The breeding plot was located in the South Coast of the Crimean Peninsula, on mild slopes of the South-West exposure, at an elevation of 123 m above the sea level. The breeding plot soils were rather heavy, clayey admixed with gravel.

The climate is mild warm Mediterranean sub-humid, characterized by a relatively small amplitude of daily and annual temperatures, with warm winters, mild hot summers and long warm autumns. The first frosts are usually registered in early December, and the last – in the middle of March. Thus, the growing season of grape begins from the first days of April finishing at the end of November. In very warm years, some late grapevine varieties retain their leaves until January.

Winter is mild, small frosts often alternate with frost-free periods. Frosts usually do not reach the level when damage of buds on annual shoots is observed. In years of extremely cold winter the temperature drops to –12…–13 °C. Therefore, even non-frost-resistant varieties do not suffer from winter frosts in the Crimea. In the second half of March, with a noticeable increase in temperature, the buds begin to swell, and in the first or second decade of April – to burst. However, temperature rises relatively slowly in April and May due to proximity to the sea. The inhibitory effect of low temperatures also affects flowering, which is usually registered in the first half of June. The beneficial effect of the sea is observed in the second half of summer and in autumn when daily and monthly temperatures do not show any violent oscillations. Autumn is warm, mild dry, with a lot of sunny days. Summer and autumn months are characterized by a relatively low amount of precipitation and air humidity.

The conditions do not favor the distribution of such diseases as downy mildew, gray rot and anthracnose. Among fungal diseases powdery mildew causes the greatest harm to vineyards, while downy mildew spreads only sporadically. The most widespread grapevine pests on the South Coast of Crimea are phylloxera and European grape moth, which produces three generations per season here.

Laboratory testing of genotype resistance to low temperatures. The laboratory method of testing frost resistance was based on the recommendations of S. Pogosyan (1974) and M. Chernomorets (1985), with some methodology modifications (Zlenko et al., 2018). In short, the diagnostics of frost resistance of grape genotypes was carried out by stepwise hardening and freezing of two-eyed cuttings of mature shoots as follows: from +8 to +4 °C for 14 days (hardening stage I); from –3 to –5 °C for 11 days (hardening stage II); and –10 °C for 1 day (hardening stage III). Then cuttings were frozen stepwise in the temperature range: from –16 to –24 °C with a 2 °C temperature change interval; from –24 to –30 °C with an interval of 10 °C. After each of ten sequential freezing stages (–16 °C for 2 days; –18 °C for 3 days; –21 °C for 2 days; –24 °C for 2 days; –25 °C for 3 days; –26 °C for 2 days; –27 °C for 2 days) 5 cuttings of each genotype were placed to refrigerator with a temperature of +2 °C for 3 days for gradual defrosting. Then cuttings were water-soaked for 1 day and placed for sprouting in half-liter containers with water at a room temperature (+22 °C).

The assessment of frost resistance was carried out according to a 9 point scale of International Organization of Vine and Wine (OIV) descriptor, with the following points of resistance: 1 – very low (–15 °С), 3 – low (–18 °С), 5 – medium (–21 °С), 7 – high (–24 °С), 9 – very high (–27 °С and lower). The degree of genotype resistance to frost stress was determined after 4 weeks of sprouting in water by assessing the percentage of shoot development from buds after each stage of freezing. For a more objective assessment of the vine shoots vitality after freezing, the length of the developed shoots, the number and length of roots, as well as the development of inflorescences were additionally determined.

Determining the resistance to pathogens in the field. Phenotypic data were obtained by evaluating plants in the field against a natural infection background without the use of fungicides

The nature and percentage of leaves damage were accounted according to generally accepted methods (Buga, 2007). Up

to 30 leaves from different parts of a plant were examined on every accounting bush. Each season, we carried out two examinations: the first was performed 3 weeks after grape flowering, the second – at the beginning of grape ripening. The percentage of leaves affection and degree of disease development on leaves were determined using the following scale: 0 – no signs of affection; 1 – single and barely noticeable spots on leaves; 2 – up to 10 % of leaf surface is affected; 3 – 11–25 % of leaf surface is affected; 4 – 26–50 % of leaf surface is affected; 5 – more than 50 % of leaf surface is affected. Disease development (R, %) for a specific genotype was calculated using the formula:

**Formula:**
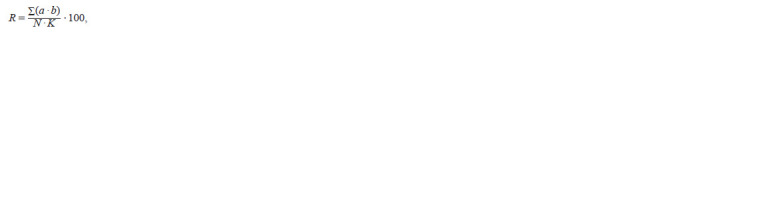
Formula

where, a is a score of the scale, according to which the lesion was evaluated in the experiment; b is the number of affected leaves within the range of this score; N is the total number of leaves evaluated (pcs); K is the highest score of the scale; and 100 is the conversion factor.

The data obtained were averaged, and then the results were interpreted according to the OIV international standards (O-452, O-454), where the degree of grape plant resistance to fungal pathogens was assessed by the degree of leaf affection using the following point scale: 1 – very low degree of resistance (extensive surface affection by the pathogen is more than 50 %); 3 – low degree of resistance (the area affected by the pathogen is 30–50 %); 5 – medium degree of resistance (the area affected by the pathogen is 20–30 %); 7 – high degree of resistance (weak pathogen affection – up to 10 %); 9 – very high degree of resistance (very small or no pathogen affection). For data analysis, the value of maximum degree of affection was used.

Laboratory testing of resistance to pathogens. In addition to the field evaluation, a phytopathological screening was carried out using the disk-test method. For the disk-test, the leaves of recombinant lines were collected in duplicate in June–July. The fourth and fifth young leaves starting from the shoot tip were taken from each hybrid plant. Grape plants, from which leaves were collected, were left unsprayed with fungicides. The disinfected leaves were placed in agar medium in Petri dishes. Visual assessment of lines resistance was carried out 6–12 days after inoculation using the OIV descriptors 452- 1 (‘Resistance degree of leaves to Plasmopara viticola in laboratory conditions (disk-test)’), 455-1 (‘Resistance degree of leaves to Erysiphe necator in laboratory conditions (disk-test)’) according to the above scale (Volynkin et al., 2021c).

## Results

Resistance of grape genotypes of hybrid populations to low temperatures

**Fig. 1.**
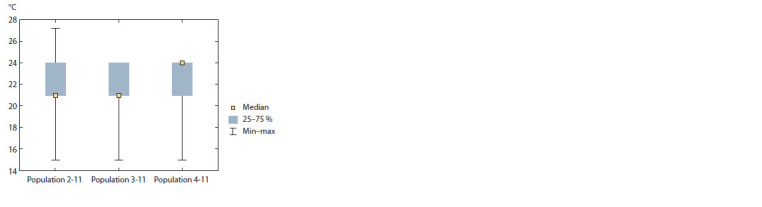
Box-and-whisker plot ref lecting variation of the trait “the lowest temperature in the experiment at which a plant survives” among the studied populations 2-11 (М. 31-77-10 × [DRX-M5-734 + DRX-M5-753 + + DRX-M5-790), 3-11 (М. 31-77-10 × 2000-305-143), and 4-11 (М. 31-77- 10 × 2000-305-163).

Frost resistance was determined in laboratory conditions in 2019. The highest range of frost resistance variation (Fig. 1) among the genotypes of the studied cross combinations with the maternal form ♀M. 31-77-10 was observed in the population 2-11 (–15…–27 °С), which reflects diversity of the hybrids with varying degrees of frost resistance and, as a consequence, provides a broad spectrum of valuable genotypes as a source for breeding. This conclusion is confirmed by the calculated breeding value (45.5 %) of this cross combination (Table 1). The population 4-11 is distinguished by a higher average degree of resistance to low temperatures, and is characterized by the highest breeding value among the studied hybrid populations (56.7 % of genotypes inherited the high level of resistance of parental forms).

**Table 1.**
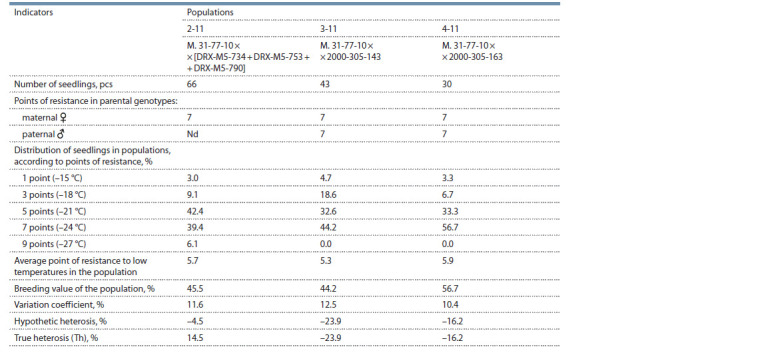
Inheritance of resistance to low temperatures by grape genotypes in hybrid populations

**Fig. 2.**
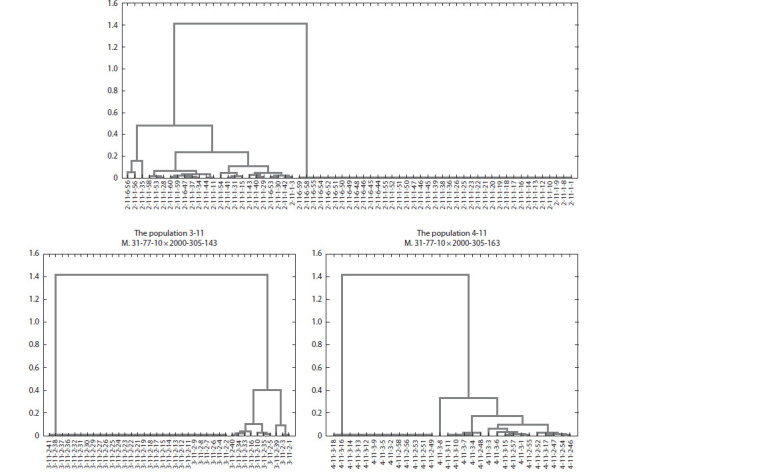
Clustering of grape genotypes according to their degree of low temperature stress resistance, observed in the populations 2-11, 3-11, and 4-11.

As a result of laboratory screening of the population М. 31-77-10 × [DRX-M5-734 + DRX-M5-753 + DRXM5- 790], about 40 % of recombinants were characterized by a high degree of frost resistance (–24 °С), and 6 % of transgressive recombinants showed a very high degree of resistance (–27 °С) (Fig. 2, see Table 1). In the populations M. 31-77-10 × × 2000-305-143 and M. 31-77-10 × 2000-305-163 (see Fig. 2), 44 and 56 % of recombinants, respectively, were characterized by a high degree of frost resistance (–24 °C).

In each studied population, there were several genotypes capable of sprouting 100 % of shoots from buds after freezing at –27 °С. In populations 2-11, 3-11 and 4-11, respectively 3, 7 and 17 % of such highly viable genotypes were discovered.

A specific combining ability was observed for each population. For example, in the combination M. 31-77-10 × 2000- 305-163, almost half of progeny (56.7 %) has high frost resistance, whereas genotypes with true heterosis were not detected (Th = –16.2). Similar principle of seedling distribution was observed in the combination of M. 31-77-10 × 2000-305-143. Hybrids of the cross M. 31-77-10 × [DRX-M5-734 + DRXM5- 753 + DRX-M5-790] were distributed almost equally into groups of medium (42.4 %) and high (39.4 %) frost resistance. Genotypes with a true heterosis effect were identified in the population (Th = 14.5) (see Fig. 2).

The resistance of grape genotypes to Erysiphe necator and Plasmopara viticola in hybrid populations

The maternal genotype ♀M. 31-77-10 is not protected by the resistance alleles in the Run1 locus and is much more affected by powdery mildew in the field compared to the paternal genotypes (e. g. 2000-305-143 and 2000-305-163) (Volynkin et al., 2021b). The percentage of oidium disease distribution on vegetative organs in the populations of recombinants varied over the years and for populations 2-11, 3-11 and 4-11 amounted to 3.2–17.1, 0.3–17.7, and 0.6–5.2 %, respectively. Due to the inheritance of resistant alleles from the paternal genome, some of the recombinant lines of hybrid populations showed a high level of resistance to E. necator (up to 26.7 %) (Table 2). Nevertheless, the average score for powdery mildew resistance among populations was lower than that observed for the paternal genotypes. The data obtained suggests that employing M. 31-77-10 as a parent in crosses with donors of resistance to E. necator allows to obtain a significant number of powdery mildew resistant genotypes in F1

**Table 2.**
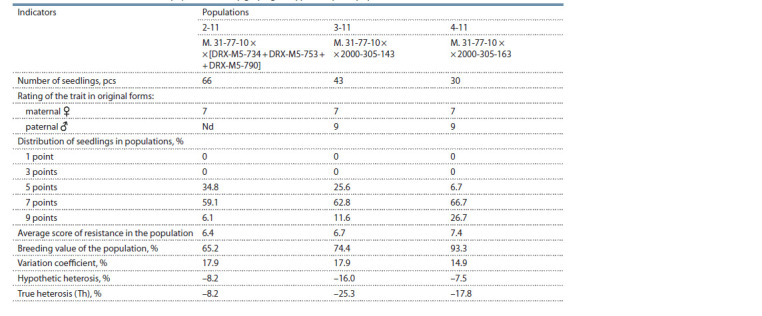
Inheritance of resistance to Erysiphe necator by grape genotypes in hybrid populations

The average scores of resistance to P. viticola established in the population 3-11 (M. 31-77-10 × 2000-305-143) and in the population 4-11 (M. 31-77-10 × 2000-305-163) were intermediate compared to parental genotypes (Table 3). The percentage of downy mildew distribution on vegetative organs in hybrid populations fluctuated in different years and amounted to 1.3–28.3, 0.2–14.8, and 0–18.6 % for populations 2-11, 3-11 and 4-11, respectively. Employing genotypes 2000-305-143 and 2000-305-163 in cross combinations as male parents allows producing 100 % sustained progeny. Remarkably, among the progeny of the cross M. 31-77-10 × [DRX-M5-734 + + DRX-M5-753 + DRX-M5-790] (population 2-11), 21.2 % of heterosis seedlings were observed to show the highest level of resistance (9 points).

**Table 3.**
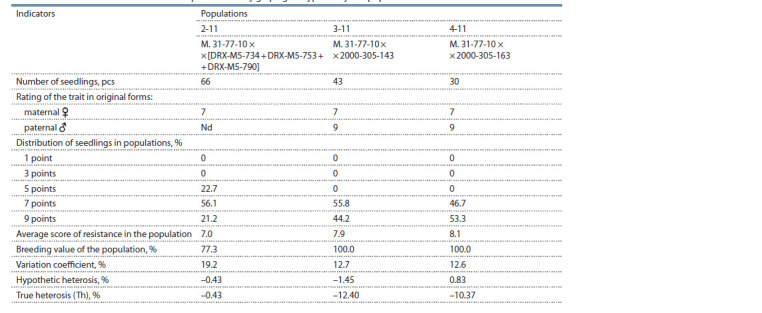
Inheritance of resistance to Plasmopara viticola by grape genotypes in hybrid populations

The obtained results of the field evaluation of resistance to pathogens were confirmed by experiments on laboratory assessment of resistance using the disk test method (Volynkin et al., 2021с). The results indicate the great importance of remote hybridization of V. vinifera with V. rotundifolia, as well as derivatives of the cv. Seibel 13666 to obtain grapevine genotypes, resistant to fungi pathogens and frost.

## Discussion

Among all grape species, the V. rotundifolia Michx. is the only one having a complex of biological properties, missing in V. vinifera L. grape varieties (Patel, Olmo, 1955).

Vitis rotundifolia is also the only native North American ancestor, the cultivated varieties of which were obtained without any genome introgression from other species of the Vitis genus, including V. vinifera. Difficulties in hybridization of genotypes of V. vinifera and V. rotundifolia are related to differences in the number of chromosomes (V. vinifera L., subgenus Euvitis, 2n = 2x = 38 chromosomes; V. rotundifolia Michx., subgenus Muskadinia, 2n = 2x = 40 chromosomes). For a long time, after such interspecific crossings attempts, breeders did not get fertile plants. The first fertile hybrid (F1) between V. vinifera and V. rotundifolia, the N.C. 6-15 hybrid (2n = 2x = 39) was obtained in the USA. Using a N.C. 6-15 hybrid, cross-pollinated with an unknown variety V. vinifera, R.T. Dunstan (1964) obtained the remote hybrid (F2) – DRX- 55 (Dunstan Rotundifolia crossing symbol) (2n = 2x = = 39). Of all remote grape hybrids, the DRX-55 was the only diploid-allotetraploid cytochimeric plant. Later on, other DRX hybrids were obtained from the same cross. By crossing remote hybrids F4 DRX-M4-520, DRX-M4-510 (n = 38) with varieties GM-35-58, Cristal and Moldova, the fifth generation (F5), combining hybrids with a somatic number of chromosomes 2n = 2x = 38, was obtained (Alexandrov et al., 1998). Among the seedlings of hybrid population F5, four synthetic genotypes were discovered, carrying a new grape genome with n = 19 (2n = 38), combining chromosomes of two species V. vinifera and V. rotundifolia.

In 2011, the pollen of three forms DRX-M5-790, -753, and -734 was kindly provided by Prof. Sh. Topale (Institute Vierul, Moldova) to the ‘Magarach’ Institute for hybridization experiments. At the same time, the hybrid genotypes 2000-305-143 and 2000-305-163 were received from Prof. R. Eibach (Federal Research Institute for Grape Breeding, Geilweilerhof, Germany). Those two genotypes were obtained by crossing French breeding line MTP3082-1-42, carrying resistance loci to powdery and downy mildew, with variety ‘Regent’. The resistance loci were originally inherited from V. rotundifolia Michx

In the USA, the Muscadine Grape Breeding Program is being developed in the University of Georgia. This is the oldest breeding program dedicated to the improvement of the muscadine grape. The UGA program began in 1909, and over the years has released over 30 cultivars. Current goals of the program include the development of new cultivars that combine large berry size with perfect flowers, earlier and later harvest dates, berries with dry stem scars and edible skins, and increased cold hardiness. The varieties can withstand frosts down to –25 °С (Morris, Brady, 2004).

Thus, V. rotundifolia can be considered as a potential donor of resistance genes to downy and powdery mildew pathogens in combination with frost resistance for breeding of new grape genotypes.

## Conclusion

Remote hybridization involving V. rotundifolia can be considered as a modern and promising trend in grapevine breeding. It opens great prospects for obtaining new forms and breeding improvement of existing varieties, expands possibilities of creating new rootstocks and enriches the gene pool of cultivated grapes. It also provides a wide range of sources for breeding and conducting in-depth cytogenetic studies to reveal general patterns of diversity formation in F1–F5 interspecific hybrids, as well as for developing research sources for grape genetics.

## Conflict of interest

The authors declare no conflict of interest.

## References

Alexandrov E., Topală Ş., Savin Gh. Crearea hibrizilor de viţă de vie
apireni Folosind formele N.C. 6-15, DRX-55, DRX-58-5. In: Book
of abstracts “Congr. II al Sociietăţii de Botanică din R. Moldova.
Biodiversitatea Republicii în preama mileniului III”. Chişinău,
1998;111

Buga S.F. (Ed.). Guidelines for Certification Tests of Agricultural Fungicides.
Nesvizh, Russia, 2007. (in Russian)

Chernomorets M.V. Resistance of a Grape Plant to Low Temperature.
Chisinau: Cartya Moldovenienasca, 1985. (in Russian)

Clark J.R., Barchenger D.W. Breeding Muscadine grapes in Arkansas,
USA: a new initiative. Acta Hortic. 2015;1082:95-98. DOI
10.17660/ActaHortic.2015.1082.12.

Di Gaspero G., Cipriani G., Adam-Blondon A.-F., Testolin R. Linkage
maps of grapevine displaying the chromosomal locations of
420 microsatellite markers and 82 markers for R-gene candidates.
Theor. Appl. Genet. 2007;114:1249-1263. DOI 10.1007/s00122-
007-0516-2.

Dunstan R.T. Hybridization of Euvitis × Vitis rotundifolia: backcrosses
to muscadine. J. Am. Soc. Hortic. Sci. 1964;84:238-242.

Gonçalves E., Carrasquinho I., Almeida R., Pedroso V., Martins A. Genetic
correlations in grapevine and their effects on selection. Aust. J.
Grape Wine Res. 2016;22:52-63. DOI 10.1111/ajgw.12164.

Ilnitskaya E., Guguchkina T., Talash A. New cold-tolerant grapevine
cultivars for red wines. Acta Hortic. 2019;1248:95-99. DOI
10.17660/ActaHortic.2019.1248.14.

Ivanisević D., Di Gaspero G., Korać N., Foria S., Cindrić P. Grapevine
genotypes with combined downy and powdery mildew resistance.
Acta Hortic. 2015;1082:47-50. DOI 10.17660/ActaHortic.2015.
1082.4.

Korbuly J. Results of breeding for resistance to winter frosts and different
pathogens using Vitis amurensis. Acta Hortic. 2000;528:
551-557. DOI 10.17660/ActaHortic.2000.528.80.

Korbuly J., Pernesz G., Pedryc A., Oláh R., Jahnke G.G. Evaluation
of frost resistance of traditional and newly bred Hungarian winegrape
cultivars. Acta Hortic. 2004;652:337-341. DOI 10.17660/Acta
Hortic.2004.652.43.

Kozma P., Jr. Evaluation of fungus-resistant wine-grape varieties. Acta
Hortic. 1998;473(473):93-104. DOI 10.17660/ActaHortic.1998.
473.9.

Likhovskoy V.V., Zarmaev A.A., Zlenko V.A., Vasylyk I.A., Rybachenko
N.A. Identification of new sources of frost resistance in grapevine
cultivars and hybrids of complex genetic structure. Magarach. Vinogradarstvo
i Vinodeliye = Magarach. Viticulture and Winemaking.
2019;21(3):186-190. DOI 10.35547/IM.2019.21.3.001. (in Russian).

Maltabar L.M., Zhdamarova O.E. On the issue of diagnosing frost resistance
of eyes in grape varieties. Plodovodstvo i Vinogradarstvo
Yuga Rossii = Fruit Growing and Viticulture in the South of Russia.
2012;17(5):79-86. (in Russian).

Morris J.R., Brady P.L. The muscadine experience: adding value to enhance
profits. Research Reports and Research Bulletins. AAES Res.
Rep. 974. 2004.

Nenko N.I., Ilyina I.A., Kiseleva G.K., Yablonskaya E.K. Low-temperature
stress tolerance of grapevine varieties of different ecological
and geographical origin. Proc. Latvian Acad. Sci. Sect. B. 2019;
73(1):56-65. DOI 10.2478/prolas-2018-0046.

Novikova L.Yu., Naumova L.G. Regression analysis of winter hardiness
of grape cultivars from Ya.I. Potapenko Don Ampelographic
collection. Magarach. Vinogradarstvo i Vinodeliye = Magarach.
Viticulture and Winemaking. 2018;4:59-61. (in Russian).

Patel G.I., Olmo H.P. Cytogenetics of Vitis: I. The hybrid V. vinifera ×
V. rotundifolia. Am. J. Bot. 1955;42:149-159.

Pavloušek P., Postbiegl E. Genetic resources of grapevine in lednice na
Morave. Acta Hortic. 2003;603:605-608. DOI 10.17660/ActaHortic.
2003.603.81.

Pogosyan S.A. Guidelines for the Selection of Grapes. Yerevan: Hayastan
Publ., 1974. (in Russian).

Polulyakh A.A., Volynkin V.A., Likhovskoy V.V. Genetic resources
of grapes of the Magarach Institute. Conservation problems and
prospects. Vavilovskii Zhurnal Genetiki i Selektsii = Vavilov Journal
of Genetics and Breeding. 2017;21(6):608-616. DOI 10.18699/
VJ17.276. (in Russian).

Read P.E., Gu S., Gamet S., Schild J. Testing of varieties and selections
under challenging climatic conditions. Acta Hortic. 2004;652:65-72.
DOI 10.17660/ActaHortic.2004.652.6.

Ricciardi V., Marcianò D., Sargolzaei M., Maddalena G., Maghradze D.,
Tirelli A., Casati P., Bianco P.F., Failla O., Fracassetti D., Toffolatti
S.L., De Lorenzis G. From plant resistance response to the discovery
of antimicrobial compounds: the role of volatile organic compounds
(VOCs) in grapevine downy mildew infection. Plant Physiol.
Biochem. 2021;160:294-305. DOI 10.1016/j.plaphy.2021.01.035.

Shen Q., Lin Y., Li Y., Wang G. Dynamics of H3K27me3 modification
on plant adaptation to environmental cues. Plants. 2021;10:1165.
DOI 10.3390/plants10061165.

Ulitin V.O., Nudga T.A. Some hereditary patterns of manifestation of
frost resistance of groups of grapes of various origin under conditions
of model freezing. Nauka Kubani = Science of the Kuban.
2008;4:38-43. (in Russian)

Vasylyk I.A., Likhovskoy V.V., Zarmaev A.A., Zlenko V.A., Rybachenko
N.A. Diagnostics of frost resistance of grape varieties in the conditions
of stress modeling. Magarach. Vinogradarstvo i Vinodelie =
Magarach. Viticulture and Winemaking. 2020;22(2):105-110. DOI
10.35547/IM.2020.17.22.004. (in Russian)

Vavilov N.I. Theoretical Foundations of Breeding. Moscow, 1987. (in
Russian)

Volynkin V.A., Gorislavets S.M., Volodin V.A., Vasylyk I.A., Lushchay
E., Likhovskoi V.V., Potokina E.K. Immunogenic breeding
program. Stage I – phytopathological screening of the grape gene
pool. In: E3S Web of Conferences. FARBA 2021. 2021с;254:03003.
DOI 10.1051/e3sconf/202125403003.

Volynkin V., Levchenko S., Poluliah A., Likhovskoi V. Models for
estimation of the existing grapevine gene pool bioversity and for
the breeding of new cultivars. Acta Hortic. 2018;1190:15-20. DOI
10.17660/ActaHortic.2018.1190.3.

Volynkin V., Levchenko S., Vasylyk I., Likhovskoi V. Analysis of
F2–F6 generations from hybridization with Vitis rotundifolia at
the Institute Magarach. Acta Hortic. 2020a;1289:269-274. DOI
10.17660/ActaHortic.2020.1289.38.

Volynkin V., Likhovskoi V., Levchenko S., Vasylyk I., Ryff I., Berezovskaya
S., Boyko V., Belash D. Modern trends of breeding cultivars
for recreational areas of viticulture. Acta Hortic. 2021a;1307:13-20.
DOI 10.17660/ActaHortic.2021.1307.3.

Volynkin V., Likhovskoy V., Polulyakh A., Levchenko S., Ostroukhova
E., Vasylyk I., Peskova I. Native grape varieties of the Euro-Asian
eco-geographical region of Russia: taxonomic, biological and agroeconomic
specificity of cultivars from Crimea. In: Vitis: Biology and
Species. New York: Nova Sci. Publ., 2020b;45-72.

Volynkin V., Polulyah A., Klimenko V., Likhovskoi V., Oleinikov N.,
Levchenko S., Pavlova I.A., Zlenko V., Kotolovets Z., Pytel I.,
Roshka N. Breeding for Ukrainian table grape varieties. VITIS –
J. Grapevine Res. 2015;54(Spec.iss.):157-158. DOI 10.5073/vitis.
2015.54.special-issue.157-158.

Volynkin V., Polulyakh A., Levchenko S. Vasylyk I.A., Likhovskoy V.V.
Aspects of the particular genetics of grapes prolonged for all horticulture
crops. In: Kossi Baimey H. (Ed.). Horticultural Crops. London:
IntechOpen, 2020с. DOI 10.5772/intechopen.90566.

Volynkin V., Polulyakh A., Levchenko S., Vasylyk I. Genome evolution
and genetic diversity of grapes. Acta Hortic. 2020d;1297:407-412.
DOI 10.17660/ActaHortic.2020.1297.54.

Volynkin V., Vasylyk I., Volodin V., Grigoreva E., Karzhaev D., Lushchay
E., Ulianich P., Volkov V., Risovannaya V., Blinova S., Alekseev
J., Gorislavets S., Likhovskoi V., Beatovic A., Potokina E. The
assessment of agrobiological and disease resistance traits of grapevine
hybrid populations (Vitis vinifera L. × Muscadinia rotundifolia
Michx.) in the climatic conditions of Crimea. Plants. 2021b;10(6):
1215. DOI 10.3390/plants10061215.

Xiaoyan L., Lianguo L., Jinyin W., Yan L., Jinli G. Introduction experiment
of the cold resistant wine grape cultivar ‘Frontenac’.
Acta Hortic. 2015;1082:61-62. DOI 10.17660/ActaHortic.2015.
1082.6.

Zlenko V.A., Volynkin V.A., Vasylyk I.A. Frost-resistance of new grape
varieties and hybrids of complex genetic structure. In: LUCRĂRI
ŞTIINŢIFICE. Chişinău, 2018;47:243-247.

